# Editorial: Qualitative research applied to public health: new topics and insight

**DOI:** 10.3389/fpubh.2024.1371938

**Published:** 2024-02-06

**Authors:** José Granero-Molina, María Dolores Ruiz-Fernández, Isabel María Fernández-Medina, Susana Núñez-Nagy, Iván Claudio Suazo Galdames

**Affiliations:** ^1^Department of Nursing, Physiotherapy and Medicine, University of Almería, Almería, Spain; ^2^Ciencias de la salud, Universidad Autónoma de Chile, Santiago, Chile; ^3^Department of Physiotherapy, University of Alcalá, Alcalá de Henares, Madrid, Spain

**Keywords:** public health, qualitative research, experiences, nursing research, health sciences research

The concept of public health refers to the science and art of preventing diseases and promoting, protecting, and improving health (1). The essential functions of public health include monitoring the health of the population, keeping watch on risk factors, guaranteeing access to healthcare services, and promoting research, among others. Although there have been many advances over the years in vaccine development, emergency response, the promotion of sexual and reproductive health, and access to medicine, a single solution has yet to be found which meets the needs of the population in different health systems (2). Classical epidemiology studies the phenomena of health and disease in the community, considering the population as a group of individuals without considering the many individual and social factors. Qualitative methodology contributes to the study of the determinants of health and disease, healthcare planning, the detection of needs, and the evaluation of interventions from the experiences of individuals and societies (3). In the face of a unitary, measurable, and external reality to the cognoscente, there is a reality which each person creates within the framework of their culture, tradition, and history. Therefore, the implementation of a dual research perspective in public health must involve quantitative approaches that address the generality of the study problem, but also qualitative ones, which include multiple layers of diversity and the range of lived experiences (4, 5). As reflected in our Research Topic, the experiences of patients, professionals, and families are key to understanding public health problems.

Research does not occur isolated from the interests and powers of the academic, scientific, personal, or theoretical field (6). Together with the technical interest in knowing the reality in order to transform it (Zang et al.), practical interest points to the intersubjective understanding of the health-disease process, and emancipatory interest points to actively taking charge of this process. In line with studying the experiences of patients, Wang et al. found specific action plans, medical feedback, and periodic records as facilitators of adherence to lifestyle prescriptions among patients with non-alcoholic fatty liver disease. Bailey-Davis et al. found that obese patients undergoing treatment in primary care expect personalized treatment options and referrals to effective community programmes. Campaña et al., suggests that being a woman with a low educational background coming from the public health system outside of the capital could contribute to barriers for effective healthcare for lung cancer in Chile. Gabay, proposes that positive experiences in the relationship between the patient and nurse instill hope for being discharged in intensive care units. Experiences of young generations on the social problems of parental care are addressed by Peng et al., emphasizing that governments should guarantee that adult children receive help to balance their work, life, and parental care responsibilities. Chen et al. found that major epidemics accelerate and promote major social changes, technological development, political, and economic measures. Experiences of members of public health associations in tracking and treating COVID-19 cases in migrants and refugees are studied by Dawson-Hahn et al.. Qualitative research also studies the experiences of evaluating public health intervention programmes, such as the case of Thoumi et al. when addressing health inequalities in Latinx communities in North Carolina; or the perspective of professionals on the barriers, facilitators, and elements for improving the +AGIL Barcelona programme (Canet-Vélez and Solis-Navarro). Teaching-learning experiences are also addressed by qualitative research in the different stages. In undergraduate studies, Zhu et al. suggests that a synthesis of knowledge and practice is needed to improve the professional skills of undergraduate nursing students. In postgraduate studies, Sánchez-Muñoz et al. found that the residency period is important in the training and acquisition of skills as a Family and Community Nursing Specialist Nurse in Spain, and improvements are needed to guarantee quality training and more visibility. Myroniuk et al. recommends involving health sciences students in public health programmes aimed at the community. Experiences of public healthcare professionals have also been studied in our Research Topic. Canet-Vélez et al. suggests that legal regulation has provided a security framework for nurse prescribing. However, strategies are needed for its comprehensive development, public acceptance and to give visibility to nurse prescribing at an international level. Along with practical interest, emancipatory interest is key in qualitative research. Researchers analyze public health problems through self-reflection, seeking social transformation, and participant involvement for change. This positioning generates empowerment, leading patients to participate in decision-making and take charge of their own health. Along these lines, Röger-Offergeld et al. studied how the participation of women as co-researchers leads to their social empowerment beyond the results of the research itself. Melhem et al. found that health literacy and empowering survivors of colorectal cancer promotes a more positive experience when interacting with healthcare systems.

Gadamer and Habermas criticize the excessively objectified and decontextualized nature of positivism and instrumental reason in 20^th^ century philosophy, recognizing the role of the subject in the creation and acquisition of knowledge and understanding (7). Qualitative research is increasingly common in contemporary health sciences, helping to incorporate the perspectives of the participants (patients, family, professionals, managers, etc.) in the design and development of the research, treating them as equals. Qualitative public health researchers could strengthen dialogue with conventional research paradigms by fostering an understanding of interdependencies (8). Investigating social determinants and health inequities requires epidemiologists and public health researchers to expand theories, research methodologies, and involve all participants (9, 10). The perspective of those who use public health services differs from the professional, clinical or academic perspective (11). Qualitative research enables an understanding of how people interpret and respond to public health policies, thus weighing the sources of academic and experiential knowledge (12). The main strength of qualitative research is the in-depth and rich descriptions of data that is studied (13). For this reason, it is used in global public health when the problems cannot be analyzed from traditional quantitative approaches, when “silenced voices” are not heard, or when sociocultural contexts are key in decision making and problem solving (14). This contribution, which complements epidemiological research, is key for all knowledge disciplines that develop research on global public health and on all communities in particular.

## Author contributions

JG-M: Writing—review & editing. MR-F: Writing—review & editing. IF-M: Writing—review & editing. SN-N: Writing—review & editing. IS: Writing—review & editing.
